# The Capacity of the Indonesian Healthcare System to Respond to COVID-19

**DOI:** 10.3389/fpubh.2021.649819

**Published:** 2021-07-07

**Authors:** Yodi Mahendradhata, Ni Luh Putu Eka Andayani, Eva Tirtabayu Hasri, Mohammad Dzulfikar Arifi, Renova Glorya Montesori Siahaan, Dewi Amila Solikha, Pungkas Bahjuri Ali

**Affiliations:** ^1^Center for Health Policy and Management, Faculty of Medicine, Public Health and Nursing, Universitas Gadjah Mada, Yogyakarta, Indonesia; ^2^Directorate for Public Health and Nutrition, Ministry of National Development Planning, Jakarta, Indonesia

**Keywords:** COVID-19, pandemic, Indonesia, healthcare, surge capacity

## Abstract

The Indonesian Government has issued various policies to fight Coronavirus Disease (COVID-19). However, cases have continued to fluctuate over a year into the pandemic. There is a need to assess the country's healthcare system's capacity to absorb and accommodate the varying healthcare demands. We reviewed the current capacity of Indonesia's healthcare system to respond to COVID-19 based on the four essential elements of surge capacity: staff, stuff, structure, and system. Currently available medical staffs are insufficient to deal with potentially increasing demands as the pandemic highlighted the human resources challenges the healthcare system has been struggling with. The pandemic has exposed the fragility of medical supply chains. Surges in the number of patients requiring hospitalization have led to depleted medical supplies. The existing healthcare infrastructure is still inadequate to deal with the rise of COVID-19 cases, which has also exposed the limited capacity of the healthcare infrastructure to manage medical waste. The COVID-19 pandemic has further exposed the weakness of the patient referral system and the limited capacity of the healthcare system to deliver essential health services under prolonged emergencies. The Indonesian Government needs to ramp up the country's healthcare capacity. A wide range of strategies has been proposed to address those mounting challenges. Notwithstanding, the challenges of increasing healthcare capacity highlight that such efforts could represent only one part of the pandemic response equation. Effective pandemic response ultimately requires governments' commitment to increase healthcare capacity and flatten the curve concurrently.

## Introduction

The Coronavirus Disease (COVID-19) pandemic has been an unprecedented test of healthcare systems worldwide, especially in low- and middle-income countries (1). There have been 160,813,869 confirmed cases of COVID-19 globally reported by WHO as of May 14, 2021 (2). COVID-19 has escalated demands for screening and testing suspected cases, contact tracing and isolation of cases, and managing severe cases in hospitals, including in intensive care units (ICUs) (3). Healthcare systems generally have not been designed to meet the demands of large-scale disasters, such as pandemics (1). Even healthcare systems in high-income countries have been overwhelmed (4, 5). Therefore, a critical aspect of a country's response is the healthcare system's ability to expand quickly to meet an increased demand for medical care, commonly referred to as surge capacity (6).

Indonesia is an important bellwether for the state of democracy in the era of COVID-19 (7). It is the world's fourth most populous nation and a Muslim-majority democracy with hundreds of ethnic groups and substantial numbers of religious minorities. Alongside its size and diversity, the country shares some common features with other populous democracies like India, Brazil, and the United States (7). The first COVID-19 cases in Indonesia were confirmed on March 1, 2020 (8). The Government has since issued various policies to fight COVID-19 (9). However, over 1 year into the COVID-19 Pandemic, cases continued to fluctuate. By June 4, 2021, Indonesia had reported 1,837,126 cases and 51,095 deaths (2). The most crowded island of Java (56.1% of the country's population) has the highest caseload, with all six provinces in the island making up around 66.1% of the national tally (10). Concerns remain as to the future trajectory of COVID-19 in the country Concerns remain as to the future trajectory of COVID-19 in the country (11). Thus, there is a need to assess Indonesia's healthcare system's capacity to absorb and accommodate increasing healthcare demands. In this article, we review the current capacity of the country's healthcare system to respond to COVID-19 based on the four essential elements of surge capacity, namely, staff (e.g., healthcare workers), stuff (e.g., supplies), structure (e.g., hospital beds and medical waste treatment), and system (e.g., referral and essential health services) (12).

## Healthcare Capacity Assessment

### Staff (Healthcare Workers)

Currently available medical staff in Indonesia are insufficient to deal with potentially increasing demands for managing COVID-19 cases. This pandemic highlighted the human resources challenges the country's health system has been struggling with, characterized by an inadequate physician-to-population ratio, an inequality of physician geographical distribution, and a significant shortage of nurses and midwives (13). The ratio of physicians to population stands at only 0.38 physicians per 1,000 population (14). The country's population of 264 million is currently served by only 1,206 pulmonologists, 4,134 anesthesiologists, 350 intensivists, 6,084 pediatricians and 1,811 clinical pathologists. Indonesia's COVID-19 rapid response task force has estimated that the country will need an additional 1,500 doctors (especially pulmonologists, anesthetists, and general physicians) and 2,500 nurses to manage the surge of COVID-19 patients (14). Furthermore, 22–26% of all active pulmonologists, internists, anesthesiologists, and radiologists work in DKI Jakarta, a province with 3% of the total population (15). The Ministry of Health (MoH), during the pandemic has recruited 2,785 volunteers that were assigned to two field hospitals and four other MoH-owned hospitals. The volunteers were general practitioners (62%) and nurses (27%). Only 24 (1%) of the volunteers were specialist doctors, and the rest were other healthcare professionals (16).

The shortage of healthcare professionals has been further aggravated by the deaths of healthcare professionals, which have been reported worldwide (17). High mortality and many infections have also been reported among physicians managing COVID-19 patients in Indonesia (18). As of May 3, 2021, the Indonesian Medical Association has notified 366 COVID-19 fatalities among physicians (19). Fatalities among Indonesia's frontline health workers are expected to remain high if no improvements are made. Lack of personal protective equipment (PPE) has been commonly cited as a cause of death among healthcare professionals globally (20, 21). Such lack of PPE, including coveralls, N95 masks, and face shields, has also been reported in Indonesia (18).

By May 15, 2021, the government had vaccinated 1,502,037 healthcare workers, covering practically all registered workers in the sector. Of that figure, 1,369,098 workers, or 93.15%, have received double doses of the CoronaVac, the COVID-19 vaccine made by the Beijing-based pharmaceutical company Sinovac (22). From January to March 2021, the MoH monitored 128,290 healthcare workers in Jakarta province who received the CoronaVac shot. The MoH officials reported that the vaccine was 94% effective in protecting the healthcare workers from symptomatic cases, a far higher figure than those of previous large-scale clinical trials (23).

With the escalating number of confirmed and suspected cases, the workload of healthcare workers in Indonesia has also been overwhelming, leading to long and irregular hours of continuous work, consequently triggering psychological distress (24). Indonesian healthcare workers with direct contact and responsibility to treat COVID-19 patients have reportedly exhibited a higher risk of experiencing depressive symptoms and burnout (25). It has been reported that 83% of healthcare workers in Indonesia experienced moderate to severe burnout (26). The study also found that 41% of healthcare workers were emotionally fatigued; 22% lost their sense of empathy; 52% lost their self-confidence. The most significant stressors among Indonesian healthcare workers are necessity of wearing PPE every day, hearing reports of new cases in the media, lack of staff, not knowing when COVID-19 will be brought under control, and feeling that there are no adequate protective measures (27). They also fear transmitting Covid-19 to their families. Coping mechanisms among Indonesian healthcare workers so far mainly encompass adopting a positive attitude; reading about COVID-19, how to prevent it, and how it spreads; following the appropriate steps for protection (mask, gown, etc.); avoiding public places to minimize exposure to COVID-19; and keeping busy at home to stay away from COVID-19 (27).

### Stuff (Supplies)

Surges in patients requiring hospitalization have led to depleted medical supplies and escalating the utilization of limited medical equipment (e.g., ventilators). Shortage of high-flow oxygen devices and mechanical ventilation, especially in small cities and outside Java, has been reported (18). Lack of PPE for healthcare workers such as coveralls, N95 masks, and face shields across healthcare facilities in the country is particularly worrying (18). Such shortage has been reported even in COVID-19 referral hospitals (28). In healthcare facilities where hazmat suits are severely limited, healthcare personnel must wear thin plastic raincoats when transporting patients under observation for COVID-19 (13). Some healthcare workers have had to reuse masks and gloves. This shortage was the worst when the public was panic buying and stockpiling medical-grade masks, hand sanitizers, and gloves, which caused the price to increase drastically (28). To better manage the limited supply of PPE and essential equipment, the MoH has also introduced guidelines for hospitals to differentiate distribution of PPE based on the level of risk of the hospital service area, i.e., red zone (COVID-19 areas) and green zone (Non-COVID-19 areas) (29).

The pandemic has exposed the fragility of medical supply chains in Indonesia. The medical supply system in Indonesia has so far relied mainly on the global supply chain. The People's Republic of China (PRC) produced approximately half the world's face masks (30). About 80% of active ingredients required for pharmaceutical compounding hail from China and India (31). Amid the COVID-19 outbreak in the PRC, travel bans, export bans, and factory shutdowns have put significant strain on PPE supply chains (32). To encourage business actors to support the procurement of the necessary medical equipment to halt the spread of COVID-19, the government has issued several regulations to relax licensing requirements for the importation and production of medical devices. The MoH has expedited the application process for the licenses required to produce domestically and distribute specific medical devices and household supplies to deal with COVID-19. It (i) has accelerated certification services for production and distribution certificates and (ii) offers a one-day service for marketing authorization (33). The National COVID-19 Task Force has distributed supplies across the provinces assisted by the Indonesian Armed Forces. When these supplies have reached the province, distribution to the health facilities is then managed by the provincial COVID-19 task force. By December 29, 2020, the government had already distributed 9.7 million PPE items, 25.1 million surgical masks, 7.8 million N95 masks, 1,310 portable ventilators, 1.1 million rapid tests, 5.8 million PCR reagents, and 3.8 million RNA reagents (34).

### Structure (Hospital Beds and Medical Waste Treatment)

The existing healthcare infrastructure in Indonesia is inadequate to deal with the increasing demands for healthcare services. Based on data from the Health Ministry website accessed on March 7, 2021, Indonesia has 2,925 hospitals with a bed capacity of 388.106 (35). This makes the ratio of hospital beds to population 1.49 beds for every 1,000 inhabitants. Such a ratio is significantly lower than neighboring economies such as Malaysia (1.9 per 1,000 inhabitants), Thailand (2.1 per 1,000 inhabitants), and Vietnam (2.6 per 1,000 inhabitants) (13). Moreover, the hospitals and bed capacities are not evenly distributed throughout Indonesia (36). Notably, five provinces in Indonesia have not even met the ratio of 1:1,000 for the number of hospital beds compared to the population. Two hundred and twenty-six districts cannot meet this ratio, and 10 districts do not even have hospitals (36). Moreover, Indonesia, at the beginning of the pandemic only had 1,910 ICUs with 7,094 critical care beds, which translates to about 2.7 critical care beds per 100,000 population, significantly lower than neighboring countries such as Malaysia (3.4 per 100,000 population), Thailand (10.4 per 100,000 population), and Singapore (11.4 per 100,000 populations) (37). Lack of adequate facilities for treating COVID-19 cases, particularly negative pressure wards and ICU rooms, especially outside Java, has been reported (18). Many non-ICU isolation wards in Indonesian hospitals are also not in line with required standards (e.g., negative pressures, no anteroom, and no protocol).

At the beginning of the pandemic, only three hospitals in Jakarta were readily designated as referral hospitals for COVID-19 patients (18). The government since then has prepared many more additional referral hospitals (including police hospitals, military hospitals, and hospitals operated by state-owned enterprises) to manage COVID-19 patients (13). In addition to COVID-19 referral hospitals, which has been established by the MoH and the local governments, the central government at the beginning of the Pandemic has opened COVID-19 Emergency Hospital in Jakarta, which occupies a building complex that used to be athletes' residences with 10,161 beds, Indrapura Field Hospital Surabaya with 242 beds, and a special hospital on Galang Island, Batam with 360 beds. Towers 4 and 5 of athletes' residences in Kemayoran were modified into self-isolation facilities. The two towers have more than 3,000 beds. Both buildings accommodate asymptomatic patients or those with mild symptoms. Towers 6 and 7 were also modified into a COVID-19 emergency hospital, equipped with 2,878 beds for patients with mild to moderate symptoms. The emergency hospital does not have critical care facilities. Patients who experience worsening of the condition must be referred immediately to the nearest COVID-19 referral hospital. Towers 8 and 9 were modified into self-quarantine facilities with a total capacity of 4,167 beds. Meanwhile, other cities with many cases have built COVID-19 emergency hospitals utilizing government buildings, sports arenas, and hotels (38).

On March 10, 2020, 132 hospitals were designated as COVID-19 referral hospitals through the Decree of the Minister of Health No. 169/2020. The MoH subsequently issued a Circular Letter requesting the directors of hospitals owned by the MoH to convert 20–40% beds from the general ward into COVID-19 wards to increase care capacity, of which 10–25% should be allocated for critical care (39). Currently, the MoH website reports 13,854 critical care beds in ICU and 6,644 in other intensive care rooms (40). The MoH reported that the bed occupancy rate for COVID-19 patients in the second week of May 2021 was around 33%. However, the current capacity and occupancy rate still varies across provinces, and in some contexts, geographical access to these hospitals persists as a significant barrier (40).

The surge of COVID-19 cases has also exposed the limited capacity of the Indonesian healthcare infrastructure to manage medical waste. Improper disposal of medical waste has been a major environmental problem in Indonesia even before the pandemic (41), when the estimated amount of medical waste generated from 2,813 hospitals was ±366 tons per day (42). The number has been estimated to be five times higher during the pandemic (41). Notably, only 82 hospitals (out of a total of 2,899) have licensed incinerators on their premises. The remaining hospitals have to contract private waste management companies, mostly (92%) operating on Java (43). Most of these third-party companies operated without proper procedures even before the pandemic (41). The risk of illegal dumping, cross-contamination, and disease transmission increases due to the extended distance from the hospital to the final disposal site (43). Laid-back regulation and weak monitoring have created loopholes in the medical waste management system.

The government has accordingly released a circular note 2/2020 to allow hospitals to operate unlicensed incinerators during the finalization stage of the permit attainment process in response to the concerns raised above (43). The government has also recommended that healthcare facilities should coordinate with industries to manage their medical waste disposal under the supervision of Provincial and District Health Offices. Within the next 5 years, the government will build 32 medical waste treatment facilities equipped with incinerator technology at several locations. Development in the five locations had begun in 2020, with a total capacity of 1,200 kilograms per hour, followed by development at six other locations in 2021 and seven locations for the 2022–2024 period. Notably, there has been a rise in the number of companies that offer hazardous waste treatment services, from only six companies in 2018 concentrated in Java to 20 companies, as of February 2021, with a total capacity of 384,120 kilograms of waste per day (44).

### System (Referral and Essential Health Services)

The COVID-19 pandemic has exposed the weakness of the patient referral system in Indonesia. In theory, the country's referral system provides a pathway for patients to be referred from primary care facilities to secondary care facilities and subsequently to tertiary care facilities (45). However, in practice, the referral system in Indonesia, even before the pandemic, has been hampered by a shortage of specialists and poorly-equipped referral facilities, as well as weak coordination. The backbone for the referral system is SISRUTE. This internet-based service connects patient data from primary health services to higher services (horizontal and vertical), which has been implemented nationally since 2016 (46, 47). By 2020, SISRUTE has been implemented in 11,388 healthcare facilities across Indonesia, including 2,962 hospitals and 7,588 primary health care centers (47).

Unfortunately, SISRUTE implementation for COVID-19 referrals has been much less than optimal, as shown by the widespread use of other referral methods (e.g., WhatsApp messaging, phone calls); the number of hospitalized patients not in line with hospitalization criteria; and lack of real-time information (47). These deficiencies are likely to be associated with challenges for implementing SISRUTE, which include healthcare providers' commitment to real-time data entry and quick response; readiness of supporting facilities and infrastructure; and lack of supervision (47). Thus, the government urgently needs to improve SISRUTE utilization by ensuring adequate resources, training, supervision, and monitoring and evaluation. The government should also explore ways to enhance the existing referral system by adopting a digital triage system (48).

The pandemic has also revealed the limited capacity of Indonesia's healthcare system to deliver essential health services under prolonged emergencies. Health workers have reported health service disruption at the community level, with closures of nearly 76% of village health posts and suspension of over 41% of home visits (49). Health workers have also reported disruptions in various services at the primary healthcare level, including family planning, immunization, and other routine maternal and child healthcare services. Reasons for suspension of services included community safety concerns, mobility restrictions, and health workers' anxiety. Immunization services, in particular, have been disrupted in more than 90% of village health posts and 65% of primary health care centers (50). The immunization service interruption has various causes, including the high risk of local transmission of COVID-19 in the catchment areas, the limited number of dedicated vaccinators who have also been diverted to respond to COVID-19, and mobility disruptions due to travel restrictions (50).

The government had issued a recommendation to postpone routine health checks to prevent older people from being exposed to COVID-19. However, older people tend to have more chronic conditions, and fewer opportunities for health checks could undermine their health status. A recent survey showed that about 11% of older people who needed to go to health facilities during the interview found difficulty doing so (51). Nearly half of the respondents answered that they were afraid of being infected with COVID-19. About a quarter of the respondents stated that the health facilities were closed or services for older people were not available. Almost one-third of the respondents who needed consultation in health facilities postponed consultations to avoid COVID-19 exposure. About 12% of the respondents experienced a shortage of routine medicine during the pandemic. Almost half of the respondents who experienced a lack of routine drugs during the pandemic stated that they did not have money to buy medicine. The following most typical reason was the closure or absence of services for older people at health facilities or pharmacies, followed by “no one takes them to buy medicines at health facilities/pharmacies” and “no stock of medicine in health facilities” (51).

Disruption in essential health services threatens progress in the achievement of health targets. Modeling of the indirect impact of the COVID-19 pandemic on maternal and newborn health in India, Indonesia, Nigeria, and Pakistan over 12 months has projected as many as 31,980 additional maternal deaths, 395,440 additional newborn deaths, and 338,760 additional stillbirths (52). Indonesia, notably, has also experienced a significant drop by 25–30% in the reported number of people diagnosed with tuberculosis (TB) between January and June 2020 as the pandemic has reduced access to TB diagnosis and treatment due to extra pressure on health services and impacts on care-seeking behavior (53).

## Discussion

Our assessment suggests that although the Indonesian Government has made considerable effort to strengthen the country's COVID-19 response, much remains to be done to improve surge capacity. While the COVID-19 pandemic is extraordinary, shortages of healthcare workers in Indonesia have been a longstanding problem. The country's physician-to-population ratio is much lower than in neighboring countries such as Vietnam (0.8 physicians per 1,000 population), Thailand (0.8 physicians per 1,000 population), and Malaysia (1.5 physicians per 1,000 population) (13). Nevertheless, shortages of healthcare workers are experienced by other countries as well. At the time of writing, India faces a severe shortage of nurses to fight the Covid-19 outbreak. India's Central Government claims it has recruited 2,206 specialist doctors, 4,685 medical officers, and 25,593 staff nurses in public hospitals across the nation under the National Health Mission since June 2020. These appointments, however, still fall short of the level the country needs (54). Workforce shortages in the UK have left the National Health Service (NHS) vulnerable to the COVID-19 outbreak. Healthcare worker shortages are a critical barrier to increasing NHS capacity and are why the Nightingale field hospitals have not been fully mobilized, despite the intense pressure on health services. The imminent risk of the NHS being overwhelmed has led to a third lockdown (55).

Thus, the Indonesian Government urgently needs to consider potentially effective human resources strategies, such as task-shifting, which have been used successfully elsewhere for chronic conditions like HIV (56). Through task-shifting, residents can be given the authority to perform tasks of pulmonologists. One ICU (Intensive Care Unit) attending could also potentially lead a team of five trained general practitioners to manage a large ICU in a COVID-19 referral hospital (57). Similar arrangements can also be considered for High Care units (HCU), Neonatal Intensive Care Units (NICU), or Pediatric Intensive Care Units (PICU) to address the limited availability of specialists. Another potential strategy is fast-tracking clinical training through an accelerated program to enable qualified personnel to enter service after a shorter intensive training period, as is implemented elsewhere for nursing (58). There have also been recommendations to consider recruiting final-year medical students to join the task force within their limits of competence (59). Meanwhile, recruiting more volunteers, especially trained nurses, and redistributing healthcare professionals within the region could optimize the allocation of existing clinical personnel. Implementation of these potential human resources strategies needs to be complemented with supervision and standing order acts to ensure effective implementation and outcomes.

Increasing mental health risks among healthcare workers handling COVID-19 patients reported in Indonesia have also been documented in other countries. A study in China reported high rates of depression (50%), anxiety (45%), insomnia (34%), and distress (72%) (60). Studies from Italy and France reported a high prevalence of depressive symptoms, post-traumatic stress disorder, and burnout (60). The long-term effect on the health of those working in healthcare remains to be seen. Thus, the Indonesian Government needs to consider potential strategies to help reduce the negative psychological responses of Indonesian healthcare workers. Resilience training (training prospectively designed to develop or enhance resilience among health professionals, e.g., the Stress Management and Resiliency Training—SMART) program has been shown to benefit healthcare workers by improving their confidence in coping with disasters (61, 62). Other strategies which have been shown to work in different settings include providing accommodation where staff could temporarily isolate themselves from their family, managing working time, and organizing psychological counselors to regularly visit rest areas to listen to difficulties or stories encountered by staff at work and provide appropriate support (63). One of the critical challenges to offer psychological support to healthcare workers is the shortage of psychologists/psychiatrists. There are only 2,500 clinical psychologists and 600–800 psychiatrists in the country, serving 260 million. The majority of them are based in Java, particularly in Jakarta (64). The triple challenges of healthcare professional shortage, physical health, and mental health could also be addressed by further harnessing telemedicine to tap into healthcare professionals in facilities not yet burdened with COVID-19 cases (13). Healthcare professionals who need to self-quarantine could also still attend to patients remotely via telemedicine. Intensive care physicians could also be deployed remotely through e-ICU solutions. Telemedicine can also be utilized for mental health education, psychological counseling services, and psychological self-help intervention systems for healthcare professionals (65).

As in virtually every country globally, Indonesia has also been seeing severe shortages of PPE and other healthcare supplies throughout the pandemic. Ensuring PPE availability for healthcare workers is critical for preventing further deaths among healthcare workers (66). Dissemination of PPE can be optimized based on risk assessments to high-contact/ transmission areas (67). This can be coupled with regular monitoring to avoid complete shortages. Additionally, there is an urgent need to improve PPE supply chains, train staff on prudent PPE use, and develop guidelines for decontamination and reuse of N95 respirators (68). The use of PPE could also be reduced for high-volume procedures with high-risk patient contact, such as throat swabbing, by adapting low-cost innovations such as swab chambers (69). The pandemic, however, has disrupted the healthcare supply chain globally. Shortages of raw materials are universal and have led to catastrophic price increases (70). Thus, the existing medical supply systems need to be enhanced to source and deliver essential commodities, including vaccines, medicines, and PPE for healthcare workers (32). The Indonesian Government needs to further increase the national production of medical supplies and equipment by encouraging domestic manufacturers of non-medical devices to reorient for the production of PPE and medical devices by further facilitating the licensing process. The government also needs to strengthen the supply chain by managing a safer level of buffer stocks for a more extended period, coupled with introducing a real-time monitoring system to allow early warning of potential shortages. Simplification of administration and bureaucracy on logistics in times of crisis is necessary to accelerate the availability of supplies in all regions. Scaled-up implementation of telemedicine could also reduce the use of already diminishing PPE by physically separating providers from patients.

Indonesia is also not the only country struggling with the challenge of medical waste. More than 20 cities across mainland China have been overwhelmed with medical waste as of March 2020 (71). The epicenter of the COVID-19 outbreak, Wuhan, generated more than 240 tons of medical waste daily during the epidemic's peak, compared to 40 tons before COVID-19 struck. India faced the same challenge, with data shared by the Central Pollution Control Board and Uttar Pradesh, Haryana, Delhi, and Rajasthan states showing that Delhi has the highest daily rate of biomedical waste. The lockdown also disrupted sewage treatment services in the UK. Traditional waste management practices such as landfills and incinerators, which have detrimental effects on the environment, replaced more sustainable measures such as recycling. The UK Environment Agency then permitted the temporary storage of waste and burning ash on sites that had not been granted permission (71). The Indonesian Government has planned to construct a provincial-based medical waste management facility, placing incinerators in five locations, which up to 2024 will be followed gradually by additional provinces that do not have direct access to a waste management facility (43). Such a plan needs to be supplemented by accelerating the development and implementation of non-incineration medical waste treatment technology (72). The government should also consider promoting the development and broader adoption of environmentally friendly PPE (73).

Indonesia also joined many other countries reporting disruption to essential health services over 1 year into the COVID-19 pandemic (74). Millions of people globally are still missing out on vital health care. Long-term care for chronic conditions, rehabilitation, and palliative end-of-life care is still severely disrupted, seriously affecting older people and people living with disabilities (75). The government urgently needs to safeguard essential health services. Modifying essential health services by implementing triage to separate patients from the risk of COVID-19 transmission could also be considered a strategy to maintain essential health services. Implementation of telemedicine should be scaled up as it allows the delivery of health services under pandemic circumstances (13). Telemedicine has been emerging as one of the most critical tools in Indonesia's fight against COVID-19, with telemedicine firms seeing a surge of service transactions during the pandemic (13). Therefore, the government should consider collaborating with these firms to support health services provision. Digital technology could also be harnessed to support virtual monitoring and evaluation of essential health services to allow early detection of service disruptions. Several hospitals that are ready with infrastructure and systems have begun to deliver services through telemedicine.

However, the legal basis for implementing telemedicine is still lacking. The Ministry of Health has issued Regulation Number 20 of 2019 on the Implementation of Telemedicine Services between Health Services Facilities. However, this regulation only addresses the telemedicine interaction between healthcare facilities. The Pandemic has prompted MoH to issue Circular Letter No. HK.02.01/MENKES/303/2020 concerning the Organization of Health Services through the Utilization of Information and Communication Technology to Prevent the Spreading of Corona Virus 2019 Disease (COVID-19) to allow physicians to provide services through telemedicine (76). The Indonesian Medical Council (IMC) has also issued KKI Regulation No. 74 of 2020 concerning the Clinical Authority and Medical Practice through Telemedicine during the COVID-19 Pandemic. These regulations grant physicians and dentists the clinical authority to provide medical services to patients using application/electronic systems in the form of telemedicine during the COVID-19 emergency (77). Provisions of the abovementioned MoH Circular Letter and IMC regulation will only remain in effect until the Indonesian Government officially declares the end of the COVID-19 state of a public health emergency. Thus, there is still a regulatory gap to advance telemedicine implementation beyond the Pandemic. Additionally, the current state of Indonesia's telemedicine services reflects the persistent challenge of the country's inequitable digital development, with a digital infrastructure that does not cover remote areas. If the government intends to harness the full potential of telemedicine, it must be accompanied by a commitment to developing digital infrastructure so that benefits would reach remote areas (75).

The Indonesian Government needs to ramp up the country's healthcare capacity. In terms of staff, the number and distribution of healthcare workers and their well-being are still areas of concern. Concerning logistics, there is still disproportionate distribution and a lack of dependence on imports. There has been commendable progress in terms of increasing critical bed capacity. However, the capacity to process medical waste safely is generally still lacking. Disruption to essential healthcare services also requires more attention. A wide range of strategies has been proposed to address those mounting challenges, e.g., ensuring standard protection, psychological and welfare support for healthcare professionals, accelerating development, and scaling up implementation of telemedicine; promote in-country production of medical technologies and supplies. Notwithstanding, the challenges of increasing healthcare capacity highlight that such efforts could represent only one part of the pandemic response equation. Boosting healthcare capacity should be coupled with efforts to flattening the curve, e.g., suppress transmission through the implementation of effective and evidence-based public health and social measures; reduce exposure by enabling communities to adopt risk-reducing behaviors and practice infection prevention and control. Effective pandemic response ultimately requires governments' commitment to increase healthcare capacity and flatten the curve concurrently.

## Data Availability Statement

The original contributions presented in the study are included in the article/supplementary material, further inquiries can be directed to the corresponding author/s.

## Author Contributions

YM conceptualized the idea, developed the outline, developed the first draft, managed the revisions, and finalized the manuscript. NA and EH developed the first draft and managed the revisions. MA, RS, DS, and PA critically revised the manuscript. All authors checked and approved the final version submitted.

## Conflict of Interest

The authors declare that the research was conducted in the absence of any commercial or financial relationships that could be construed as a potential conflict of interest.
